# Sonochemical co-deposition of antibacterial nanoparticles and dyes on textiles

**DOI:** 10.3762/bjnano.7.1

**Published:** 2016-01-04

**Authors:** Ilana Perelshtein, Anat Lipovsky, Nina Perkas, Tzanko Tzanov, Aharon Gedanken

**Affiliations:** 1 Bar-Ilan University, Department of Chemistry, Bar-Ilan Institute of Nanotechnology & Advanced Materials, IL-52900 Ramat-Gan, Israel; 2 Universitat Politècnica de Catalunya, Group of Molecular and Industrial Biotechnology, Rambla Sant Nebridi 22, Terrassa 08222, Spain; 3 National Cheng Kung Univ, Department of Materials Science & Engineering, Taiwan 70101, Taiwan

**Keywords:** antibacterial nanoparticles, coating stability, dyes, sonochemical coating

## Abstract

The sonochemical technique has already been proven as one of the best coating methods for stable functionalization of substrates over a wide range of applications. Here, we report for the first time on the simultaneous sonochemical dyeing and coating of textiles with antibacterial metal oxide (MO) nanoparticles. In this one-step process the antibacterial nanoparticles are synthesized in situ and deposited together with dye nanoparticles on the fabric surface. It was shown that the antibacterial behavior of the metal oxides was not influenced by the presence of the dyes. Higher *K*/*S* values were achieved by sonochemical deposition of the dyes in comparison to a dip-coating (exhaustion) process. The stability of the antibacterial properties and the dye fastness was studied for 72 h in saline solution aiming at medical applications.

## Introduction

The preferred technique for coating with nanoparticles (NPs) in most scientific and industrial examples is the direct impregnation of textiles in the reactant solution. Other methods such as chemical vapor deposition (CVD) of silver NPs on textiles have also been used [1]. Among the various other coating techniques the sonochemical immobilization was carried out on a large variety of substrates. Functional nanoparticles (NPs) were deposited on polymers [2], glass [3], metals [4], textile [5–6] and even paper [7], imparting to the solid substrate the properties of the immobilized particles. These include magnetic, catalytic, fluorescing, antibacterial, and antibiofilm properties. The sonochemical coating technique guarantees a very good adherence of the deposited NPs to the substrate resulting from the high speed (>500 m/s) at which the NPs are thrown at the substrate by microjets created after the collapse of the acoustic bubbles near a solid surface [8]. The excellent adherence is reflected in the case of nano-CuO coated on cotton, which after 65 intensive washing cycles at 75 °C in a hospital washing machine, still maintained their bactericidal effect, yielding a reduction of about log 5 after this long process. Moreover, SEM pictures demonstrated that the CuO NPs remained on the surface [9].

Over the years, we developed two modes of NPs deposition: i) the in situ deposition mode, where the sonochemically formed NPs are thrown onto the surface subsequent to their synthesis [5–6], and ii) the “throwing stones” mode used when the desired NPs cannot be prepared sonochemically in situ. In the latter case commercial NPs are introduced in the solvent and the ultrasonic waves are employed to “throw” them as “stones” and immobilize them onto the substrate [10]. For both methods very homogeneous coating on the substrate surface is achieved irrespective of the nature of the substrate.

On the other hand, simultaneous deposition on solid surfaces of two materials was applied using methods such as reactive electron beam deposition [11], pulsed laser deposition [12], Langmuir–Blodgett [13], electrochemistry [14], ion sputtering [15], casting [16], and sonoelectrochemistry [17]. We could, however, find only one report in which sonochemistry was used for the co-deposition of AgNPs and AgCl on TiO_2_ NPs to form Ag@TiO_2_ and Ag/AgCl@TiO_2_ [18]. The synthetic process revealed that in fact it was a one-step process combining Ag^+^, TiO_2_ NPs, and NaCl in ethylene glycol solution. The sonochemical process led to the partial reduction of Ag^+^ to metallic Ag, while AgCl was also formed due to the presence of Cl^−^.

In the current work we describe the deposition of two different functional materials on textiles, e.g., dyes and biocidal NPs that were synthesized and embedded onto the surface from a solution containing precursors. Colored antibacterial textile/NPs composites were generated.

The production of textiles on which dyes and antibacterial NPs are co-deposited is normally achieved in a two-stage process. For example, Tabatabee et al. reported on the sonochemical synthesis of CdS and subsequent coating/dyeing of the textile by impregnation [19]. Niu has reported on another sonochemical attempt to impart dyes and biocidal agents on wool [20].

In the current paper we describe the simultaneous deposition on cotton fabrics of Reactive Orange 16 (RO16) or Reactive Black 5 (RB5) with antibacterial CuO or ZnO nanoparticles (NPs) from an aqueous solution. The solution contains both the dye and the corresponding M(CH_3_COO)_2_ (M = Zn or Cu) precursor, which undergoes hydrolysis under alkaline conditions (ammonia) to form ZnO or CuO. The cotton was colored with the dye and showed good antibacterial properties. The color fastness was evaluated by immersing the coated/dyed cotton in water and monitoring the absorbance of the colored fabric. Two major issues were solved in this research: i) the antibacterial activity of the metal oxide (MO) NPs was maintained while deposited simultaneously with the dye, ii) a stable sonochemical coloration of cotton fabric was achieved in spite of the rich literature on the use of ultrasonic waves for bleaching color from textiles [21–24].

## Experimental

### Water-based synthesis of metal oxide (MO) NPs and their simultaneous coating with a dye on cotton

The coating/dyeing process was carried out in a 120 mL flask containing 0.022 g of the corresponding M(CH_3_COO)_2_ precursor and 0.16 g of RO16 or RB5. A 10 × 10 cm^2^ sample of a cotton bandage was introduced in the flask filled with 100 mL of the above solution. The sonication was conducted at 30% amplitude of a 750 W booster sonicator (Sonics and Materials instrument, Ti-horn, 20 kHz). When the solution reached a temperature of 60 °C, 25% aqueous ammonia solution was added drop wise to adjust the pH to 8, and the sonochemical process was continued for another 60 min. The temperature of 30 °C was maintained constant during the sonochemical coating reaction by cooling the flask with cold water. The coated fabric was first washed thoroughly with water to remove traces of ammonia, then with ethanol, and dried under vacuum. A control experiment, in which only the dye (RO16 or RB5) was deposited sonochemically on cotton, was conducted using the same experimental conditions as described above but without the addition of M(CH_3_COO)_2_.

### Characterization of the coated fabrics

The particle morphology and size distribution have been studied with a high-resolution scanning electron microscope (HRSEM) Quanta 200 FEG from FEI (USA). The Cu and Zn concentrations on the fabric surface were determined by inductively coupled plasma optical emission spectroscopy (ICP-OES) analysis (Horiba ULTIMA 2 spectrometer) after their dissolution from the fabric with 0.5 M HNO_3_. The dyes adsorbed on the cotton were characterized by UV spectroscopy (CARY 100 Scan UV spectrometer covering a wavelength range from 300 to 800 nm). An attempt to prove the existence of the dyes on the cotton was carried out by solid state nuclear magnetic resonance (NMR). The experiments were performed on a Bruker Advance III 500 narrow-bore spectrometer, using a 4 nm double-resonance magic angle spinning (MAS) probe. ^13^C CP-MAS experiments were carried out at a spinning rate of 8 kHz, using a 2.5 ms ^1^H 90° pulse, 2 K data points, 1 K scans and a 2 ms ramped-CP period. Proton decoupling using the SPINAL composite pulse sequence at a field of 100 kHz was used during acquisition and a 3 sec recycle delay between acquisitions. Chemical shifts were given with respect to adamantane (38.55, 29.497 ppm). In addition, the ^1^H NMR measurements of the dye-containing solution was performed after the sonication to probe for possible chemical changes in the dye structure. The crystalline structure of the metal oxides was examined by XRD (Bruker D8).

### Leaching experiments

Possible leaching of the MO and dyes from the coated textile was tested in saline solution (0.9 % NaCl). A piece of fabric (70 mg) was placed in 10 mL saline and kept at 40 °C for 72 h under agitation at 100 rpm. The leaching solutions were analyzed by ICP-OES for the presence of ions. The release of NPs into the saline was examined by a high-resolution transmission electron microscope (HRTEM), JEOL, operated at 200 kV. The difference in colorization of the textiles before and after leaching experiments was characterized by reflectance measurements.

### Antibacterial test

The antibacterial activity was tested according to the procedure described by our group previously [25]. Briefly, the antibacterial activity of MO- and MO/dye-coated fabrics was tested against *E. coli*. Overnight cultures of the bacteria were transferred into a nutrient broth (NB) medium (“Difco” Detroit, MI) and grown at 37 °C with aeration. When the cell number reached 10^6^ CFU, the cells were harvested by centrifugation and washed twice with a 0.85 % NaCl solution at pH 6.5 (saline). The fabric (2 × 2 cm^2^) was placed in a vial (*d* = 2.5 cm) containing 2 mL of bacteria in saline. The bacterial suspensions were incubated for up to 60 min at 37 °C with agitation (170 rpm). Aliquots (100 μL) were taken at different time intervals (0, 7, 15, 30 and 60 min) and plated on nutrient agar plates after 10-fold dilution in saline. The plates were allowed to grow overnight at 37 °C and the viable bacteria were counted thereafter.

## Results and Discussions

### Optimization of the co-deposition

The deposition of the two compounds, the antibacterial ZnO or CuO, and the RO16 or RB5 dyes were carried out by dissolving the corresponding M(CH_3_COO)_2_ precursor and the dye in water. Basic hydrolysis of the acetates was performed by adding ammonia solution to obtain MO. A 10 × 10 cm^2^ piece of fabric was introduced in the reaction cell and the sonicator was operated for 60 min. MO NPs were formed and thrown simultaneously with the dye towards the fabric surface. The dyeing occurs as a result of supplying ultrasound energy to the solution. When high-intensity ultrasound is applied to the aqueous solution of organic molecules, these molecules are adsorbed on the surface of the sonochemically formed acoustic bubbles. When the implosive cavitation collapse occurs, many molecules are brought together to form a nanoparticle. Such a nanoparticle consists of a very high amount of desired molecules [26–27]. In the current case, the molecules of the dye that are presented in the solution form the nanoparticles, in addition to the MO that are synthesized as a result of hydrolysis reaction of metal acetate. The coating is an in situ process which takes place subsequently to the formation of the nanoparticles. High-speed jets that are generated due to the bubble collapse, throw the newly created NPs of MO and the dye, at high speed toward the textile surface where they remain strongly embedded.

**Figure 1:**
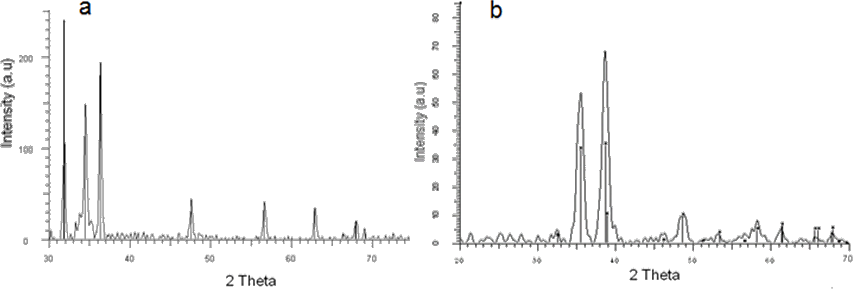
XRD patterns of sonochemically coated fabrics with: (a) ZnO and (b) CuO NPs.

Indeed, the XRD patterns of the fabric at the end of the reaction revealed the formation of MO NPs on the surface (Figure 1). The XRD pattern of the sonochemically prepared ZnO NPs correspond to hexagonal phase of zincite (Figure 1a). The peaks at 2θ = 31.772, 34.420, 36.256, 56.602, and 62.858°, are assigned to the (100), (002), (101), (110), and (103) reflection planes, respectively (PDF: 01-089-1397). The pattern of the sonochemically prepared CuO NPs (Figure 1b) refers to a base-centered monoclinic tenorite phase (PDF: 01-089-2529). The peaks at 2θ = 35.56, 38.74, and 48.74° are assigned to (−111), (111) and (−202) reflection planes. No peaks of impurities were detected. The amount of MO coated on the fabric was determined by ICP (Table 1) as described in the experimental section. The concentration of the metal acetates and the dyes was varied and the best reaction parameters, which resulted in the highest antibacterial activity and coloration are reported in the experimental section.

**Table 1:** Absorbance and ICP data of the various samples.

sample	wavelength (nm)	intensity of coating (*K*/*S*)^a^	content of oxides on bandage (wt %)

**RO16**			
RO16 sonochemically coated	500 ± 5	2.0	–
RO16 applied by dipping	500 ± 5	1.7	–
RO16 + ZnO simultaneously coated	500 ± 5	2.4	0.9
RO16 + CuO simultaneously coated	530 ± 5	1.5	0.6

**RB5**			
RB5 sonochemically coated	595 ± 5	4.5	–
RB5 applied by dipping	595 ± 5	3.2	–
RB5 + ZnO simultaneously coated	595 ± 5	4.8	0.9
RB5 + CuO simultaneously coated	540 ± 5	1.9	1.3

^a^*K*/*S*: color strength value, i.e., the Kubelka–Munk relationship, where *K* is an absorption coefficient and *S* is a scattering coefficient; *K*/*S* is a function of dye concentration on the surface.

**Figure 2:**
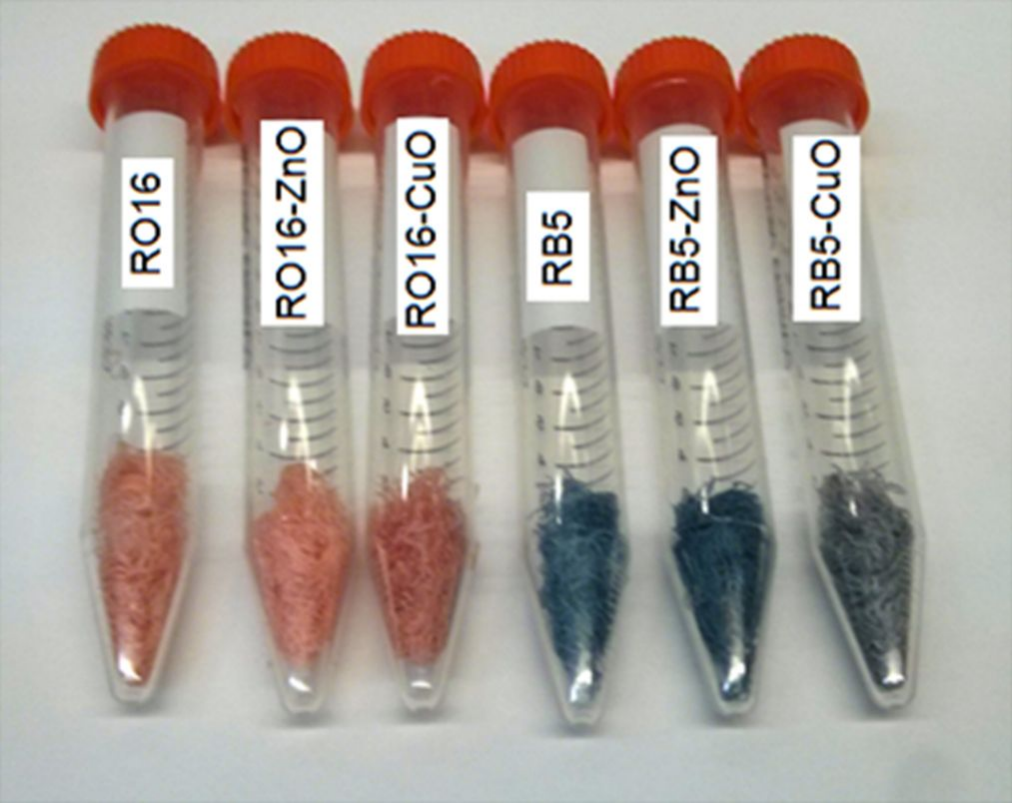
Images of textile fibers (shredded bandages) colored with RO16 and RB5 dyes and functionalized with ZnO and CuO NPs in a one-step sonochemical process.

**Figure 3:**
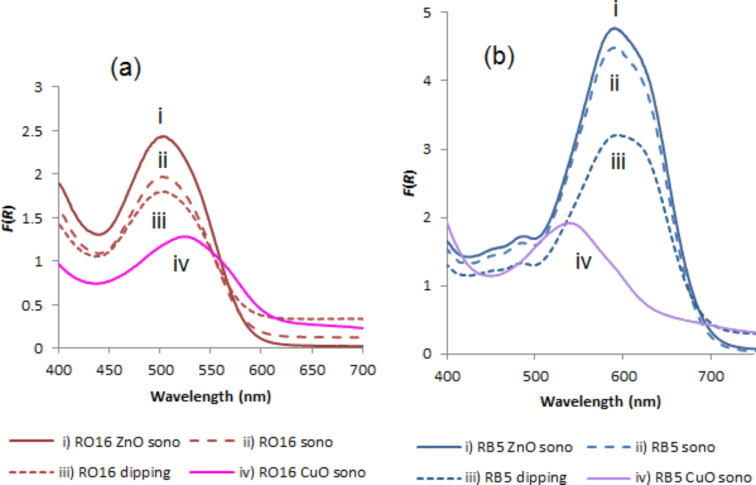
Absorption measurements in the visible region of the colored bandages coated with dyes RO16 (a) or RB5 (b) and ZnO or CuO NPs: (i) bandages coated with corresponding dyes and ZnO NPs by the sonochemical method; (ii) bandages coated with only dyes by sonochemical method; (iii) bandages coated with only dyes by dipping procedure; (iv) bandages coated with CuO NPs by the sonochemical method.

The presence of the dye on the fabric after the sonochemical reaction can be easily observed by the naked eye (Figure 2). While the color of RO16 changed only slightly when simultaneously coated with CuO, the color of RB5 changed from blue to green-blueish when simultaneously coated with CuO. These changes are reflected in the energies of the absorption peaks of RO16–CuO where a 30 nm red (1132 cm^−1^) shift is detected, while for RB5–CuO a 55 nm blue shift (1712 cm^−1^) is observed (Table 1, Figure 3). The the dye–ZnO complexes did not reveal any spectral shifts for both dyes. It is clear that CuO is interacting with the dyes, while ZnO does not show the same interaction. The reason for the CuO shifts is related to the visible absorption of CuO implying that the energy levels of CuO are in the vicinity of those of the dyes. Such an interaction is not expected for ZnO, which absorbs in the UV range.

It is worth noting that the wavelength of the absorption maximum of the dyes did not change upon sonication indicating that the deposited molecule did not undergo chemical changes. A control dyeing was carried out by dip-coating the fabrics in an alkaline (ammonia) dye solution for 60 min, the same time as the sonochemical coating reaction. Higher *K*/*S* values were obtained for the sonochemically ZnO-coated samples as compared to the regular dip-coated samples (Table 1, Figure 3). Conventional dyeing with reactive dyes is normally carried out in the presence of high amount of salts and at elevated temperatures (about 80–90 °C). Avoiding the use of salts and decreasing the process temperature while obtaining better dyeing results, is another advantage of the sonochemical vs conventional dip (exhaustion) dyeing.

### Morphology of the coating

**Figure 4:**
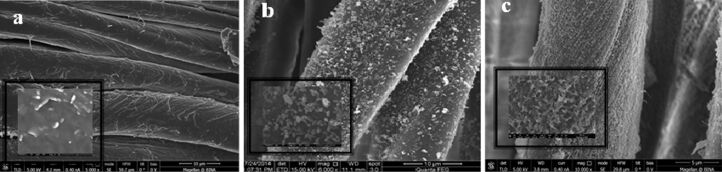
HRSEM images of (a) RB5 sonochemically deposited on cotton, (b) ZnO coated in the presence of RB5, (c) CuO coated in the presence of RB5; insets were taken in higher magnification.

The sonochemical technique appears as an efficient method for coating of substrates and textiles in particular [6,10]. This is reflected in the retention of the antibacterial properties of the coated fabrics even after 65 washing cycles at 75 °C [9]. Herein, for the first time we report on the co-deposition of two functional materials. The morphologies of the dye alone and co-deposited dye and metal oxides were studied by HRSEM and are shown in Figure 4. The morphology of RB5 sonochemically deposited on cotton is presented in Figure 4a. Under ultrasound irradiation the dye molecules form NPs which are deposited onto the surface of the textile. The creation of organic nanoparticles from their solution by sonochemical method was previously described by our group [25–26]. The HRSEM images of the fabric coated with ZnO and RB5 is presented in Figure 4b, and the fabric coated with CuO and RB5 is illustrated in Figure 4c. The average size of ZnO–RB5 NPs coated on the cotton fabric is ca. 150 nm and the exhibit prolate spheroidal shape. The CuO–RB5 particles have a needle structure with ca. 80 nm length and 10 nm width. Identical morphologies, needles and prolate spheroidal-shaped are observed for the RO16 coated with both metal oxides.

### Chemical structure of the immobilized dyes

**Figure 5:**
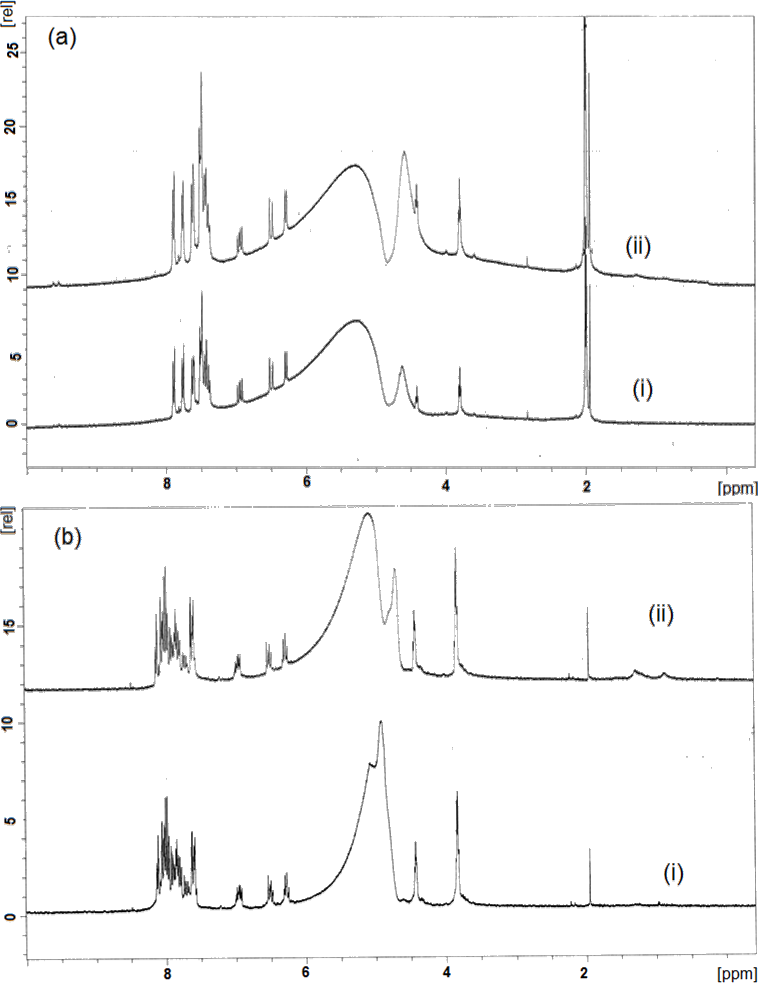
^1^H NMR spectra of the dyes solution of (a) RO16 and (b) RB5 before (i) and after (ii) the sonochemical treatment.

In order to study the influence of the sonication procedure on the dyes in the solution, each of dyes (RO16 and RB5) was dissolved in D_2_O at a concentration of 1.6 g/L and sonicated for 60 min. The ^1^H NMR spectra were measured, and no changes were observed in the NMR spectra of the solutions before and after sonication, confirming that no degradation of the dyes has occurred during the sonochemical treatment (Figure 5). The spectra of RO16 before and after the sonochemical treatment looked identical, and the small difference in the spectra of RB5 in the broad signal around of 5 ppm was attributed to the residual of the peaks resulting from the solvent suppression.

Additionally, the coated/dyed bandages were characterized by solid state ^13^C NMR. The reflectance spectra of cotton sonochemically coated with RO16 and RB5 were compared with the spectrum of untreated cotton. The solid state NMR spectra did not reveal any signal of the dyes. Apparently, the small amount of dye deposited on the fabric is below the detection limit of the NMR measurement.

### Antibacterial activity

**Figure 6:**
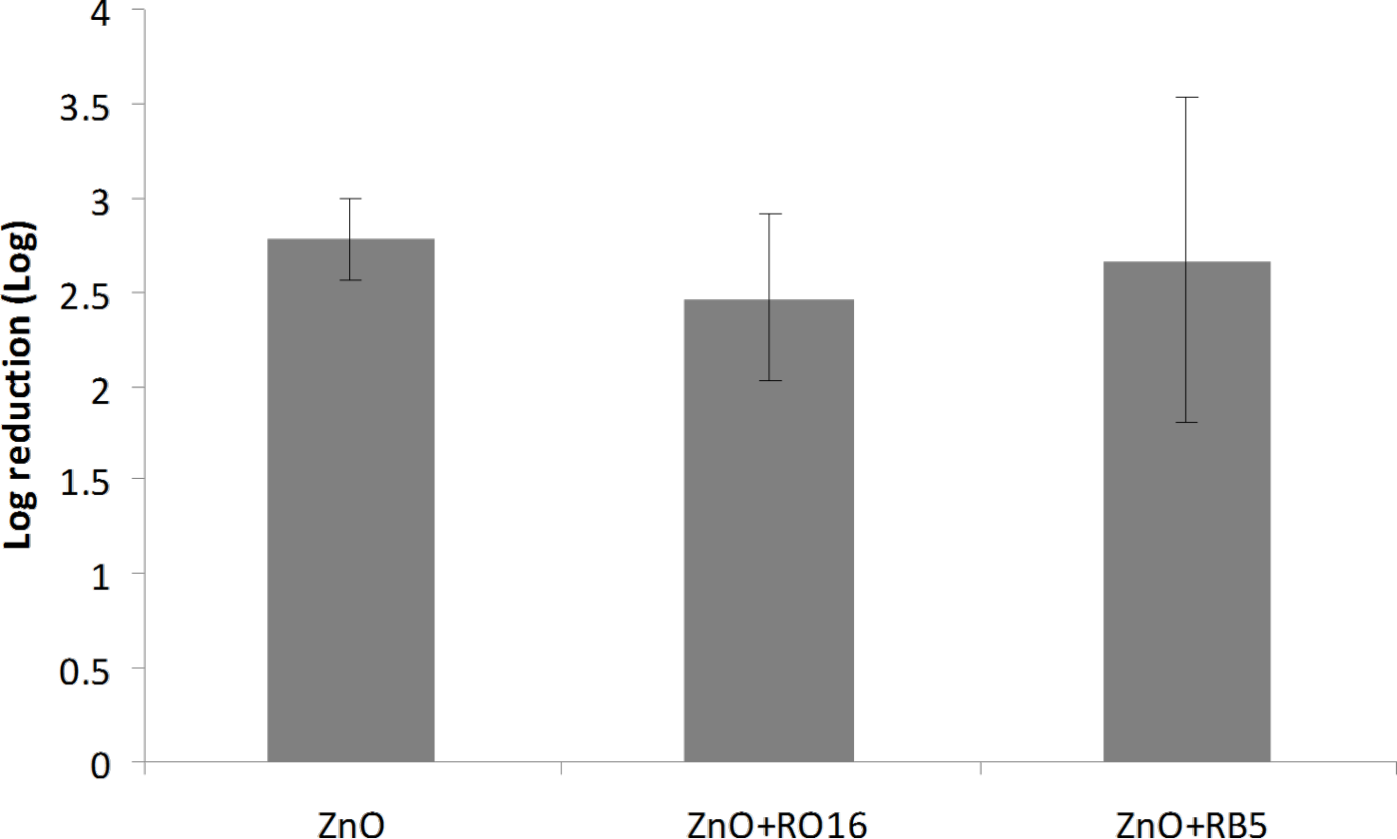
Antibacterial properties of ZnO-coated bandages and ZnO/dye-coated bandages.

**Figure 7:**
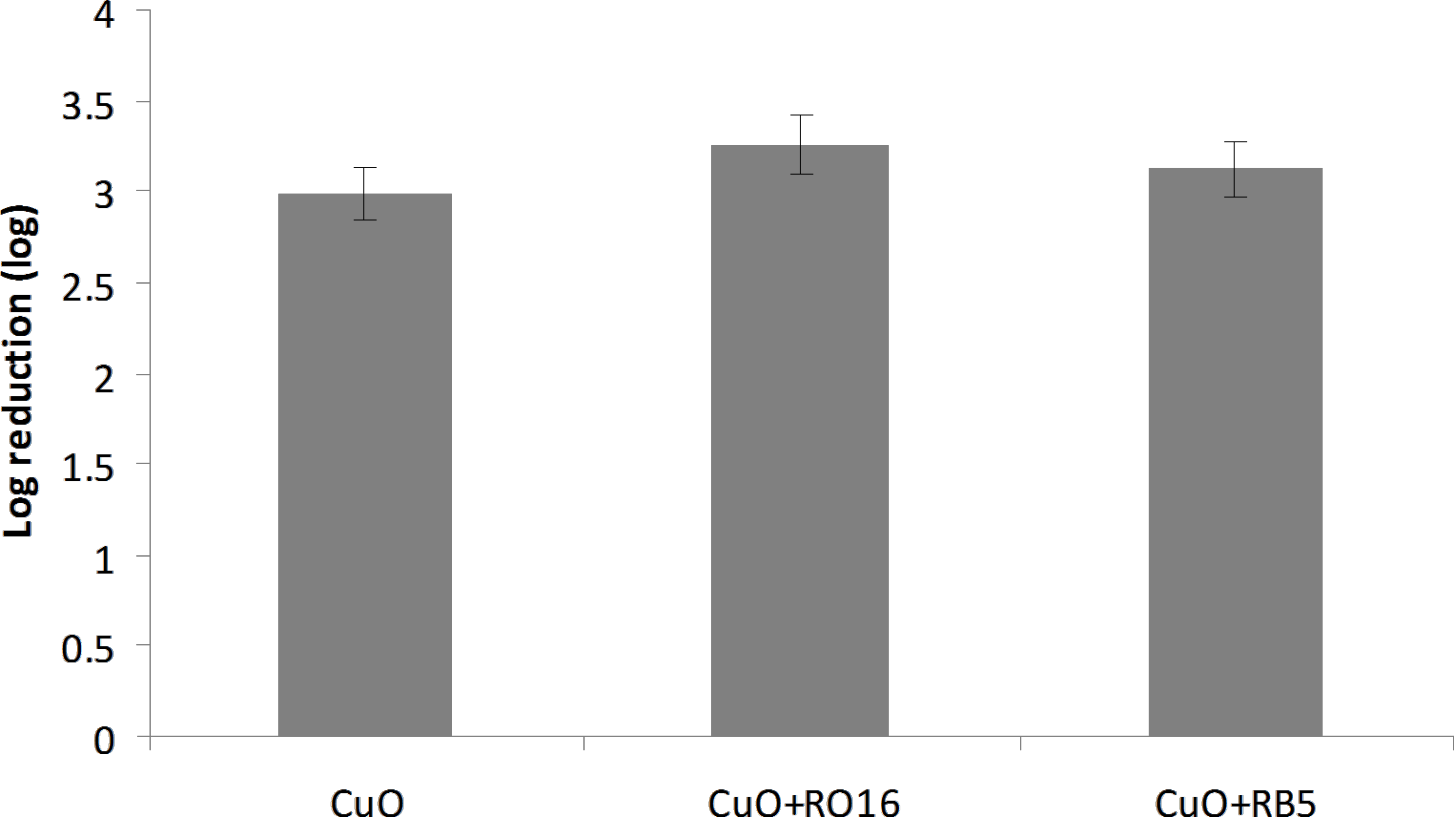
Antibacterial properties of CuO-coated bandages and CuO/dye-coated bandages.

The antibacterial properties of sonochemically produced MO NPs synthesized from ethanol/water solution were previously evaluated and reported [6,10]. In the current paper, the antibacterial properties of entirely water-based synthesized MO NPs complexed with the dye are presented. The antibacterial properties of the ZnO and CuO were first evaluated against *E. coli*, and compared with co-deposited dye/MO. Two and a half log reduction was obtained in 1 h for the ZnO coated bandage. The addition of RO16 or RB5 slightly reduced the antibacterial activity of ZnO NPs (Figure 6). The activity of CuO bandage was significantly higher and a 3 log reduction was observed already after 15 min of incubation, while after 30 min a 4.5 log reduction was achieved (Figure 7). The addition of RB5 or RO16 did not affect the activity of CuO NPs.

### Stability of coating

One of the key factors of classifying the quality of coating technique is the stability of the active phase while exposed to liquids. In the current research, the stability of sonochemically deposited MO and dye was evaluated by soaking the coated textiles in saline solution at 40 °C for 72 h. There are a number of species that can be released from the coated surface, such as: a) nanoparticles of MO, b) metal ions, and c) dye molecules. It is known that the solubility of MO is derived from their *K*_sp_ values. *K*_sp_ of ZnO and CuO are 10^−11^ and 10^−20^, respectively. The presence of ions in the leaching solution was monitored by ICP-OES measurements. The second column in Table 2 represents the percentage of leached metal oxides NPs relatively to the initial amount of coating on the surface. In order to monitor if NPs were leached off the surface, a drop of leaching solution was placed on a copper grid and subjected to HRTEM measurements. The results did not reveal the presence of NPs on the grid, indicating that nanoparticles are not released from the coated surface, confirming their strong adherence onto the surface.

**Table 2:** Comparative studies of fabric coating based on absorption and ICP-OES data.

sample	initial content of oxides on bandage (wt %)	release of oxides into leaching solution (%)	intensity of coating (*K*/*S*)^a^	intensity of coating after 72 h (*K*/*S*)^a^	relative decrease of color intensity

**RO16**					
RO16 sonochemically coated	—	—	2.0	0.4	5.0
RO16 applied by dipping	—	—	1.7	0.2	8.5
RO16 + ZnO simultaneously coated	0.9	12.2	2.4	0.7	3.4
RO16 + CuO simultaneously coated	0.6	4.6	1.5	1.0	1.5

**RB5**					
RB5 sonochemically coated	—	—	4.5	3.7	1.2
RB5 applied by dipping	—	—	3.2	1.9	1.7
RB5 + ZnO simultaneously coated	0.9	5.4	4.8	3.9	1.2
RB5 + CuO simultaneously coated	1.3	2.5	1.9	1.3	1.5

^a^Color strength values.

A total loss in the range of 2.5–12.2% of the metal oxides was found. This loss is due to the dissolution of the M^2+^ and O^2−^ ions. As ZnO is more soluble in water, higher percentage of released ions was found for the ZnO coating.

**Figure 8:**
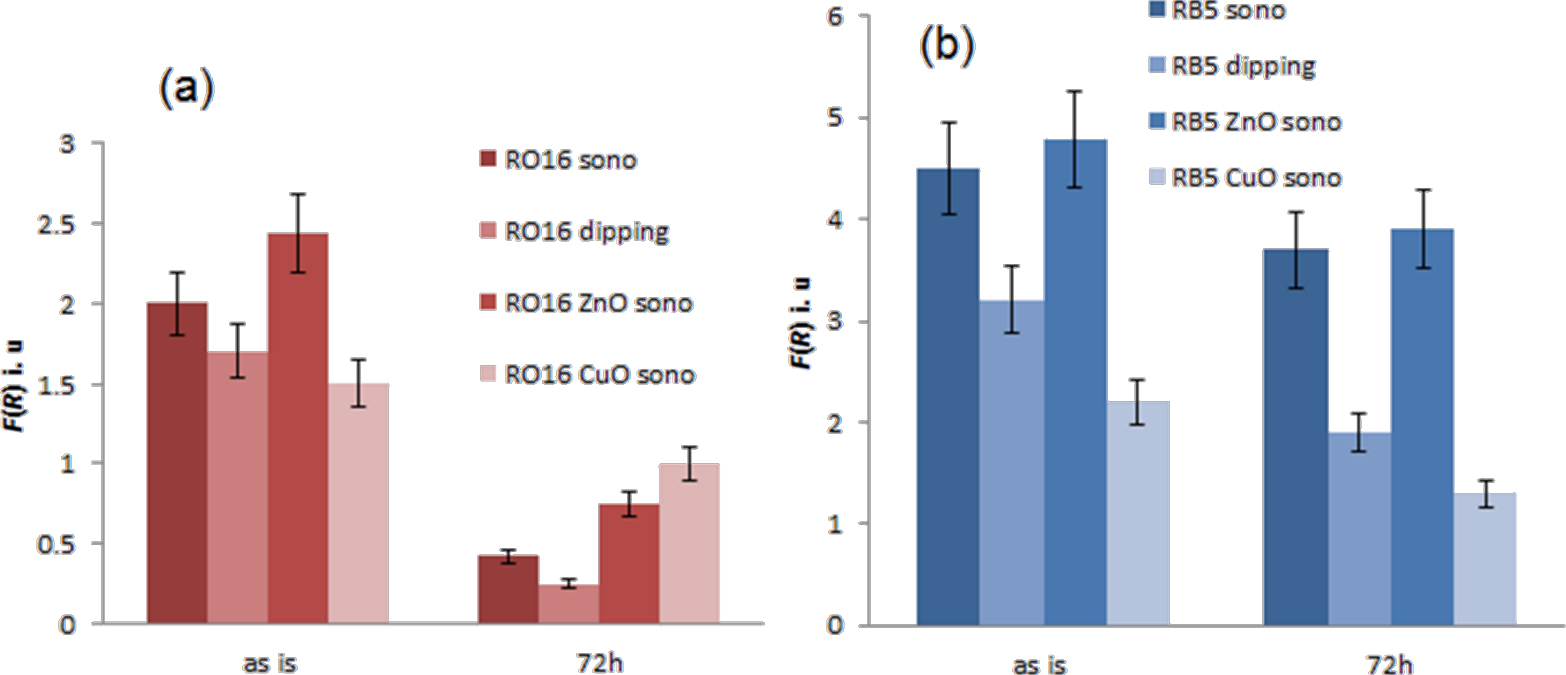
Comparison of dye stability after leaching in saline solution for 72 h at 40 °C and shaking at 100 rpm for the colored bandages coated with RO16 (a) or RB5 (b) and ZnO or CuO NPs. The data is for three independent experiments.

To follow the leaching of the dye into water and saline solution the color difference of the textiles before and after leaching experiments was studied by measuring their absorption spectra. The results are presented in (Table 2 and Figure 8). When RO16 was sonochemically coated with metal oxide, the decrease in the intensity of color was smaller in comparison to the leaching observed for the sonochemically deposited dye alone. This might indicate a possible interaction between the MO and the dye during the co-deposition. In the case of RB5, the release of the dye itself is very low and was not influenced by the presence of MO. The color intensity of the sonochemically dyed fabrics was higher than the dip-coated bandages for both dyes based on the *K*/*S* values. In addition, the color loss for the dip-dyed samples is more noticeable than for sonochemically coated dye. The sonochenical technique appears to be more efficient for dyeing of textiles compared to the conventional dyeing process. The sonochemical co-deposition of antibacterial nanoparticles and the dye impart two functions in a one-step process that led to stabilization of the dye on the surface while exposed to the solution.

## Conclusion

This is the first time that the sonochemical coating technique was applied for co-deposition of two functional materials. The simultaneous coating of the antibacterial NPs with dye imparts the textiles with antibacterial properties in addition to colorization. The antibacterial behavior of the metal oxide was not influenced by the presence of the dye. In addition, the sonochemical coating of the dye alone from its water solution was also presented. The superiority of the sonochemical coating technique over the regular dipping procedure was demonstrated. The advantages include the reduced temperature, the elimination of the use of salts, and the higher stability at wet conditions. The foreseen applications of the colored and antibacterial fabrics are in medical uses, military clothing, work-wear uniform, and as household decorative textiles.
